# Whole exome sequencing reveals pathogenic variants in *CNGA3*, *CACNA1F*, and *RPGRIP1* in consanguineous Pakistani families with diverse retinal phenotypes

**DOI:** 10.1371/journal.pone.0327176

**Published:** 2025-07-30

**Authors:** Jahangir Khan Tareen, Hamid Khan, Shamsul Ghani, Saeed Khan, Bakhtawar Khan, Yurong Wu, Muhammad Ajmal Khan, Syed Shahab Ud Din Shah, Abrar Hussain, Mubin Mustafa Kiyani, Shahid Bashir, Atta Ur Rehman, Muhammad Imran Shabbir, Hong-Tao Li

**Affiliations:** 1 Molecular Biology and Bio Interfaces Engineering Lab, Department of Biological Sciences, Faculty of Sciences, International Islamic University Islamabad, Islamabad, Pakistan; 2 Institute of Brain Science and Brain-inspired Research, Shandong First Medical University & Shandong Academy of Medical Sciences, Jinan, China; 3 Shandong Institute of Brain Science and Brain-inspired Research, Jinan, China; 4 The Second Affiliated Hospital of Shandong First Medical University & Shandong Academy of Medical Sciences, Taian, China; 5 Sandman Provincial Hospital Patel Bagh, Quetta, Balochistan,; 6 Institute of Brain disorders, Department of Physiology, Dalian Medical University Liaoning Provence China,; 7 Department of Chemistry, the Hong Kong University of Science and Technology, Hong KongChina; 8 School of Medicine, university of Maryland, Baltimore, United States of America; 9 Department of Biochemistry, Faculty of Biological Sciences, Quaid-I-Azam University, Islamabad, Pakistan; 10 Shifa college of Medical Technology, Shifa Tameer-e-Millat University, Islamabad, Pakistan; 11 Department of Neuroscience, King Fahad Hospital, Dammam, Saudi Arabia; 12 Department of Zoology, Faculty of Biological and Health Sciences, Hazara University, Mansehra, Pakistan; Odense University Hospital, DENMARK

## Abstract

This study investigates the genetic basis of retinal diseases in four consanguineous families from Pakistan, focusing on mutations in the *CNGA3, CACNA1F*, and *RPGRIP1* genes that are implicated in retinal dysfunctions such as achromatopsia, congenital stationary night blindness, and retinal dystrophies. We identified pathogenic variants in these genes, including the novel missense mutation c.955T > C; p.Cys319Arg in CNGA3 (Family 1), the frameshift mutation c.1443dupT; p.Ile482Hisfs*6 in CNGA3 (Family 2), the missense mutation c.2254G > A; p.Val752Met in *CACNA1F* (Family 3), and the frameshift mutation c.2789dupT; p.Pro931Thrfs*3 in *RPGRIP1* (Family 4). Clinical features associated with these mutations include nystagmus, photophobia, reduced visual acuity, and color vision deficiency, with some patients progressing to complete blindness. The findings were validated through Sanger sequencing, segregation analysis, and in silico prediction tools. Additionally, molecular dynamics simulations were conducted to assess the impact of the *CNGA3* p.Cys319Arg mutation on protein structure, revealing significant alterations in protein conformation and dynamics. These results highlight the significance of *CNGA3, CACNA1F*, and *RPGRIP1* in retinal health and provide valuable insights into the genetic underpinnings of retinal disorders. Our findings contribute to improved genetic counseling, potential targeted therapies, and a deeper understanding of inherited retinal diseases.

## Introduction

Recent innovations in genomic approaches have allowed for the discovery of new genomic entities and gene variants associated with a wide range of hereditary [18] and inflammatory diseases in ophthalmology [20]. Retinal function and phototransduction disorders are just some of the conditions that can be influenced by these variations, and whose genetic underpinnings are of particular interest because of the clinical relevance in terms of vision [24]. Oculocutaneous albinism (OCA) is a rare genetic disorder characterised by reduced melanin production in the skin, eyes, and hair, and associated visual disabilities [7]. This disorder emphasises the significance of genetic elements in retinal action and photoreceptor activity. OCA is a collective term for a spectrum of genetic conditions with different severities, often culminating in substantial visual dysfunction [22].

In retinal diseases, genes involved in phototransduction and retinal function are essential for preserving normal vision. Genetic mutations have been noted in patients with congenital nystagmus, a disorder characterised by involuntary eye movements [15]. This condition, commonly linked to the malfunction of photoreceptors in the retina, emphasises the importance of studying the exertions of visual signal-transmission genes [1]. The *CNGA3, CACNA1F*, and *RPGRIP1* genes are the most well-known from our list, and this article discusses their photoreceptor function and implications in retinal diseases [3,9].

The *CNGA3* gene encodes a protein subunit that forms part of a heterotetrameric cyclic nucleotide-gated channel, essential for cone phototransduction. These channels, located in the plasma membrane of cone photoreceptors, open in response to the binding of cyclic GMP (cGMP), allowing the influx of cations and subsequent depolarization of the cell [17]. The *CNGA3* gene provides instructions for making the alpha subunit of a protein called the cone photoreceptor cyclic nucleotide-gated (CNG) channel, which is required for a process known as phototransduction in the retina [5]. Mutations in *CNGA3* are thought to impair this process, resulting in achromatopsia, an unusual autosomal recessive condition that features deficient cone photoreceptor function, poor vision, nystagmus, and photosensitivity [5]. The consequences of *CNGA3* mutations extend beyond mere color blindness, impacting the overall visual experience and adaptation to varying light conditions, with the severity of symptoms often correlating with the specific nature and location of the mutation within the gene [10].

The *CACNA1F* gene encodes the alpha-1F subunit of a voltage-dependent calcium channel needed to rapidly influx calcium ions into retinal cells. Active nucleotide transport is essential for retinal signaling and cellular viability. Mutations in *CACNA1F* have also been associated with a range of retinal diseases, such as Congenital Stationary Night Blindness type 2(2) and Aland Island Eye Disease(3), as well as X-linked cone-rod dystrophy(4) (Mihalich et al. 2022) [11]. We focused on this gene because of its importance in calcium signaling, a key process in photoreceptor function and visual processing.

The *RPGRIP1* gene encodes for a protein that interacts with RPGR, an essential component of the cilium in photoreceptor cells. It is responsible for providing transport of proteins and molecules needed to maintain structure and function in the photoreceptor (Megaw, Soares, and Wright 2015) [12]. As such, mutations in *RPGRIP1* can result in several ocular conditions, including retinal dystrophies as well as retinal conditions such as Leber congenital amaurosis (LCA) and cone-rod dystrophies, and progressive vision loss (Torii et al. 2023) [13]. Mutations in *RPGRIP1* are a significant cause of inherited retinal diseases [14]. Clinical manifestations of RP are highly variable, even among individuals with the same genetic mutation [8]. Ceration affect the neuronal network, with different vitreoretinal interface impairment [16].

Pilot studies showed that *CNGA3, CACNA1F*, and *RPGRIP1* were associated with important retinal processes like phototransduction, calcium signaling, and photoreceptor structure maintenance, and were therefore selected. Mutations in these genes lead to various retinal diseases, so studying the potential role of these genes will provide more insight into the genetic cause of retinal dysfunction and retinal disease. The detailed clinical manifestations and listed in Table 1

**Table 1:** Ophthalmic features of the patients in the subject families.

Clinical Features	Family1:CNAG3:c.955T > C;p.Cys319 > Arg				Family 2: CNAG3:c.1443dup; p.Ile482Hisfs*6		Family3:CACNA1F:c.2254G > A; p.Val752Met		Family3: RPGRIP1:c.2789dupT; p.Pro931Thefs*	
Patients	V:1	V:3	V:5	V:6	IV:1	IV:2	IV:2	IV:3	IV:1	IV:6
Nystagmus	+	+	+	+	+	+	+	+	+	+
Light Sensitivity	+	+	+	+	+	+	+	+	+	+
Achromatopa	–	–	–	–	+	+	Not specified	Not specified	+	+
Loss of vision	+	+	+	+	–	–	Not specified	Not specified	+	+
Poor eyesight	+	+	+	+	+	+	Not specified	Not specified	+	+
Complete blindness	–	–	–	–	–	–	Not specified	Not specified	–	–

## Methodology

### Genetic analysis

This study involved four families with congenital ocular disorders from the Khyber Pakhtunkhwa and Balochistan provinces of Pakistan. The Ethical Review Board Committee of the International Islamic University, Islamabad, Pakistan approved the study (No. IIU (BI and BT/FBAS- 2018- 3598)) in accordance with the Declaration of Helsinki. This manuscript does not include any identifying images or personal details of participants. Ethical approval and written informed consent were obtained from all participants included in this study. Genetic testing was conducted with written informed consent from all relatives. An ophthalmologist carried out clinical histories and examinations.

We collected blood samples from subjects with RMS, healthy siblings, and parents. Genomic DNA was isolated using the salting-out method. The A 260/A/A 280 ratio was determined using a NanoDrop spectrophotometer (Thermo Fisher Scientific) to evaluate the quality of the DNA, ensuring that the ratio was within the acceptable range (1 1.7–2 2.0), indicating a high degree of DNA purity. DNA integrity was assessed by gel electrophoresis to rule out degradation.

The Illumina TruSight One clinical exome sequencing panel was used in all affected individuals to perform next-generation sequencing (NGS), as previously described [16]. This panel spans 4,800 genes linked to clinical phenotypes, including those responsible for Oculocutaneous Albinism (OCA). The mismatch between the DNA to RNA conversion sequence reads and the human genome reference sequence (hg 19) for identifying base pair changes was examined using the CLC Sequence Viewer (https://www.qiagenbioinformatics.com/products/clc-sequence-viewer/) and Chromas Lite (http://technelysium.com.au/wp/chromas/) software.

Variants discovered through NGS in genes of interest were subjected to multiple filtering criteria. Minor Allele Frequency (MAF) from the 1000 Genomes Project was applied to approximate pathogenicity as previously described [21].

For variants and segregation analysis validation, allele-specific primers were designed using Primer 3 Pulse web software. The sequences of oligos can be provided upon request. The allele-specific primers performed PCR in these studies on all affected and healthy individuals under standard conditions. The products were sequenced by Source BioScience Life Sciences (https://www.sourcebioscience.com/).

## Molecular dynamics simulation

To explore the impact of the known *CNGA3* mutation, we performed molecular dynamics (MD) simulations. The wild-type protein structure was retrieved from RCSB PDB (PDB ID: 7RHS, https://www.rcsb.org/structure/7RHS) and the mutant structures were derived from the wild-type structure using the built-in function in Maestro (Schrödinger). Protein preparation utilized the Protein Preparation Wizard, which assigns bond orders and adds hydrogen atoms, disulfide bonds, and other molecule parts as needed.

A water box (10 Å x 10 Å x 10 Å) was added using System Builder to model the physiological environment, and water molecules were included from the SPC water model. Physiological saline conditions were mimicked by using 0.15M NaCl concentrations, and additional Na+/Cl—ions were added to neutralize the protein’s charge.

Equilibration was conducted before the production run: the protein-water-ion system underwent energy minimization to prevent steric clashes. The equilibrated structure following minimization was obtained using the NPT (number of particles, pressure, and temperature) ensemble at 300 K and 1.01325 bar for 100 ns with the Desmond molecular dynamics engine. All simulations utilized the OPLS3e force field from the Schrödinger Suite 2020−2.

## Principal component analysis (PCA)

PCA was performed to assess conformational changes in CNGA3 during MD simulations. Subsequently, PDB files were generated for the simulation frames,as previously described. TM-align was utilized to align all frames to the first frame of the wild-type *CNGA3* simulation. Coordinates for the C-alpha atoms were extracted using Biopython, and PCA was implemented with the scikit-learn library. PC1 and PC2 explain X% of the variance in the protein’s movement during the simulations, providing an overview of the most significant conformational changes observed.

## Dynamic cross-correlation matrix

A dynamic cross-correlation matrix (DCCM) was calculated to evaluate the motion correlation between residues. This matrix identifies positive and negative correlations in residue movements, which is critical for understanding the protein’s internal communication network. Using the coordinates of the C-alpha atoms, the correlation coefficient between residues C(i,j)C(i,j)C(i,j) was calculated with the following formula:


$$C(i,j)=\frac{<\Delta r_{i}\cdot \Delta r_{j}>}{<{\left|\Delta r_{i}\right|}^{2}{>}^{\frac{1}{2}}<{\left|\Delta r_{j}\right|}^{2}{>}^{\frac{1}{2}}}$$

where $\Delta r_{i}$ is the displacement vector of the ith residue and the angle brackets represent the average values over all frames of the simulations. C(i,j)>0 and C(i,j)<0 indicate positive and negative correlations, respectively. The above calculation was conducted using the dccm function in Bio3D.

## Results

We identified four pathogenic variants in the families enrolled, each in a different gene. Two variants were novel, while the other two were previously reported. Two variants were found in the same gene (*CNGA3*), with the remaining two in different genes (*CACNA1F* and *RPGRIP1*). Two variants were stop gain mutations, while the others were missense mutations. Detailed clinical manifestations and comprehensive details are listed in Tables 1 and 2, respectively.

**Table 2:** In silico prediction for the pathogenicity by using different tools.

Family 1: *CNAG3*:c.955T > C;p.Cys319 > Arg						
Prediction Tools	**CADD**	**GERP**	**PolyPhen-2**	**Mutation Taster**	**PROVEAN**	**SIFT**
Score	24.8	5.13	0.999	0.999	−6.36	0.01
Prediction	High	Conserved	Damaging	Disease Causing	Deleterious	Deleterious
Family 2: *CNAG3*:c.1443dup;p.Ile482Hisfs*6						
Score	35.0	5.47	1.000	1.000	−10.24	0.00
Prediction	Very high	Conserved	Damaging	Disease Causing	Deleterious	Deleterious
Family 3: *CACNA1F*:c.2254G > A;p.Val752Met						
Score	21.8	4.83	0.843	0.999	−4.33	0.23
Prediction	High	Conserved	Damaging	Disease Causing	Deleterious	Tolerant
Family 4: *RPGRIP1*:c.2789dupT;p.Pro931Thefs*						
Score	34.9	5.58	1.000	1.000	−10.53	0.00
Prediction	Very high	Conserved	Damaging	Disease Causing	Deleterious	Deleterious

### Family 1

#### Clinical features.

Family 1 resided in Yar Hussain, District Swabi, Khyber Pakhtunkhwa. The affected individuals exhibited bilateral nystagmus, which was inherited as an autosomal recessive disorder in the pedigree. The pedigree displayed a five-generation family with four affected siblings, three females and one male. Symptoms included nystagmus, photophobia, and progressive vision loss. Genetic studies of eight individuals, two males, including one affected and six females, including three affected

### Genetic findings

In family 1, exome sequencing was performed on the proband. All variants resulting from exome data analysis were filtered using Minor Allele Frequency (MAF) of less than 0.01, from the 1000 Genomes database. Genotypes were set to homozygous, and mutation types included frame-shift, in-frame, stop gain, start loss, and missense mutation. The filtered results revealed 108 genes, among which a search identified *CNGA3* on chromosome 2. *CNGA3* is known to play a role in the eye and is associated with causing eye anomalies. the specfic *CNGA3* variant identified was located at chr2; 99,012,588T > C; c.955T > C; p.Cys319Arg in Exon 8. This variant has been previously reported in a study linked to Achromatopsia, a condition characterized by color blindness, reduced visual acuity, and nystagmus [19]. The presences of this variant in individuals with similar phenotypes suggest its pathogenic role in disrupting normal cone cell function. The parents and one nunaffected daughter were found to be heterozygous (carrier) (C/T), one daughter was homozygous (T/T) wild type and four affected individuals including one male and three females were homozygous for the mutation (C/C). Sanger sequencing confirmed the presence of the mutation, and its pathogenicity was further validated using various prediction tools (Table 2).

### Family 2

#### Clinical details.

Family 2 was recruited from Charsadda, Khyber Pakhtunkhwa, Pakistan. The parents were consanguineously married and showed no clinical symptoms. The family included four siblings, two affected (one male, one female) and two unaffected siblings (both female). The main clinical symptoms observed in the affected individuals were color vision deficiency (achromatopsia), photophobia, reduced visual acuity, and nystagmus. The family history confirmed that the phenotype was present from birth. For genetic studies, eight individuals were included, five of whom were family members: four females and one affected male.

#### Genetic findings.

In Family 2, we identified a homozygous frameshift mutation at c.1443dupT; p.Ile482Hisfs*6 in the *CNGA3* gene. This mutation results in premature termination of the transcript. The *CNGA3* gene is associated with Achromatopsia (MIM # 216900), which is characterized by color blindness, reduced visual acuity, and nystagmus. The variant was found in a homozygous state in both affected individuals and in a heterozygous state in both parents. The mutation was confirmed through Sanger sequencing, and its pathogenicity was assessed using various prediction tools. The presence of this variant in the affected individuals and its segregation within the family strongly supports its role in the observed phenotype, linking the *CNGA3* variant to the clinical symptoms of achromatopsia, light sensitivity, poor eyesight, and nystagmus.

#### CNGA3 genotype-phenotype correlation.

The *CNGA3* c.1443dupT; p.Ile482Hisfs*6 variant is responsible for the observed clinical features, which include color vision deficiency (achromatopsia), photophobia, impaired visual acuity, and nystagmus in Family 2. The demonstration of homozygosity for this frameshift mutation in the two affected individuals, along with heterozygosity in the two carrier parents, further provides evidence that this mutation is associated with the clinical phenotype. This frameshift mutation leads to the premature truncation of the *CNGA3* protein, which is critical for cone photoreceptor function, resulting in the initial presentation of achromatopsia. The family segregation pattern, in which the affected family members have inherited the homozygous mutation while the parents are heterozygous, provides strong evidence linking this alteration in the gene to the disease phenotype.

Members of Family 1 were also found to have *CNGA3* c.955T > C; p.Cys319Arg mutations associated with comparable phenotypes, including nystagmus, photophobia, and reduced visual acuity. The segregation of the mutation with the phenotype was confirmed, as it was present in affected individuals and absent in the unaffected ones. This missense mutation disrupts normal *CNGA3* protein function associated with the phototransduction cascade and, as such, contributes to the development of achromatopsia in this family. The *CNGA3* genotype correlating closely with the phenotype of achromatopsia in Family 1 also provides additional evidence for an association between this variant and the presence of clinical symptoms.

### Family 3

#### Clinical features.

Family 3 was collected from District Mastung, Baluchistan, Pakistan. This is a consanguineous family spanning multiple generations. The family has three affected individuals with varying clinical manifestations. The parents of the affected siblings are cousins and have a history of the disease on the father’s side. The affected individuals exhibit sensitivity to light, poor eyesight, and nystagmus. Family history confirmed this phenotype is congenital with a presumed X-linked inheritance pattern.

#### Genetic findings.

In Family3, we identified a missense variant at ChrX: 49,069,046C > T; c.2254G > A; p.Val752Met in the *CACNA1F* gene. The mutation was confirmed through Sanger sequencing, and upon familial co-segregation study, the variant showed a strong genotype-phenotype correlation. For example, the proband’s Father (III-7) was found to be hemizygous wild type (C/-), the Mother (III-6) was heterozygous (C/T) carrier, and one normal daughter (IV-5) was wild type (C/C). Both affected male siblings were found to be hemizygous for the pathogenic mutant type (C > T). Therefore, the variant was consistent with an X-linked inheritance pattern based on the pedigree. Various online prediction tools indicated that the identified variant is likely pathogenic.

### Family 4

#### Clinical features.

Family 4 was identified in Pishin, Balochistan. It comprises two affected individuals (IV:1 and IV:6) and normal parents in a four-generation pedigree. The affected individuals include a male (IV:6) and a female (IV:1). For genetic studies, five individuals were included, three males (III:2, IV:7, and IV:6) and two females (III:1 and IV:1). III-5 blood was included. This family is consanguineous with no history of ocular disorders. The main clinical features in affected individuals are sensitivity to light, nystagmus, and gradual loss of eyesight leading to complete blindness around 20.

#### Genetic findings.

**Fig 1:**
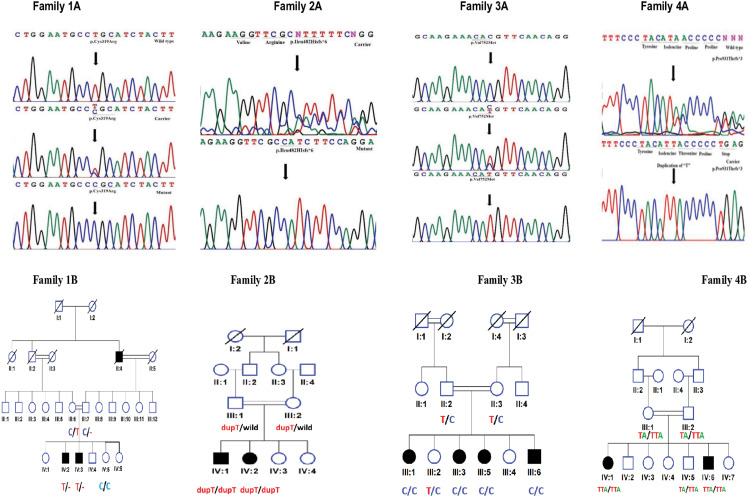
Family 1A: Chromatogram of family 1, Family 2A: Chromatogram of family 2, Family 3A: Chromatogram of family 3, Family 4A: Chromatogram of family 1. Family 1B: Pedigree and segregation of family 1, Family 2B: Pedigree and segregation of family 2, Family 3B: Pedigree and segregation of family 3, Family 4B: Pedigree and segregation of family 4.

**Fig 2:**
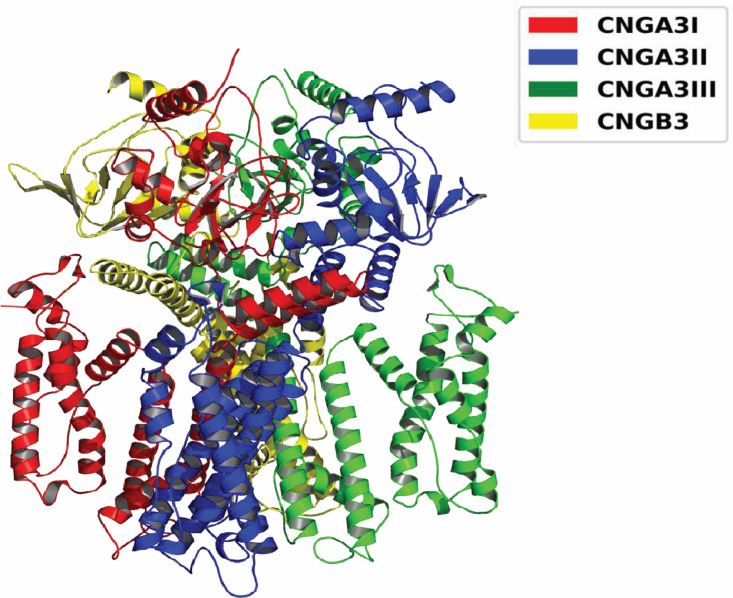
The cryo-EM structure of the CNGA3/CNGB3 channel comprises three CNGA3 subunits (*CNGA3I*, *CNGA3II*, and *CNGA3III*) and one CNGB3 subunit.

**Fig 3:**
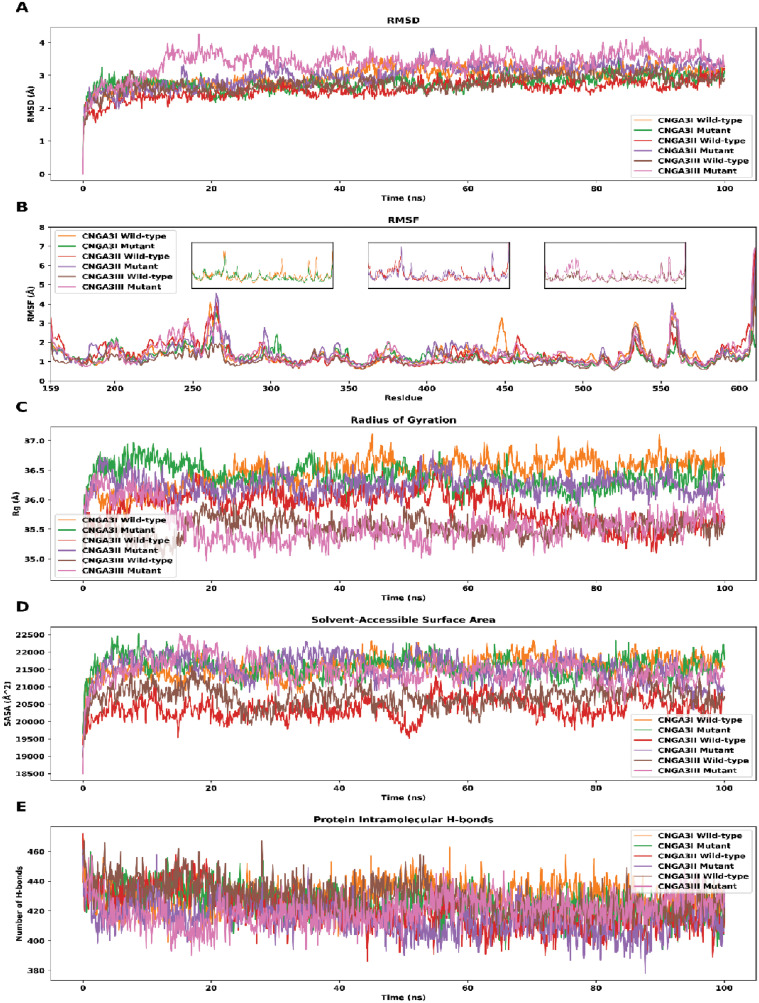
RMSD (A), RMSF (B), Rg (C), SASA (D), and protein intramolecular H-bonds (E) of wild-type and mutant *CNGA3I*, *CNGA3II*, and *CNGA3III.*

**Fig 4:**
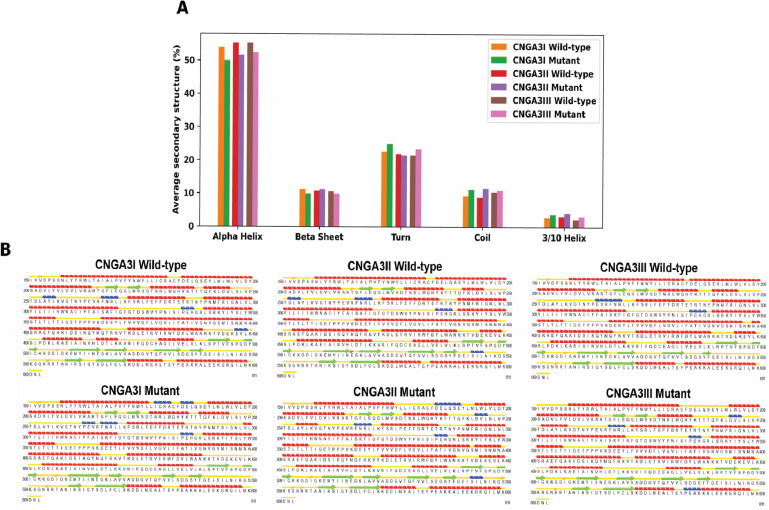
A. The percentage of average secondary structures of wild-type and mutant *CNGA3I*, *CNGA3II*, and *CNGA3III.* B. The distributions of various secondary structures in different domains of the wild-type and mutant *CNGA3I*, *CNGA3II*, and *CNGA3III*. The mutated residue (Cys319 or Arg 319) is marked red. The distributions were calculated based on the average structures of the protein after 20 ns of the simulations.

**Fig 5:**
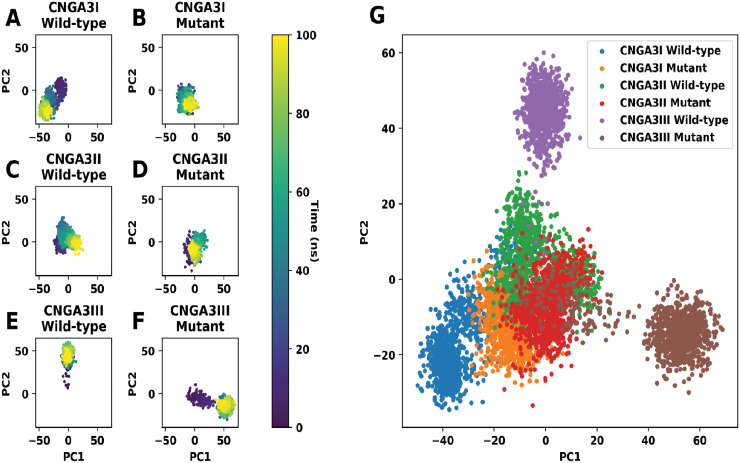
PCA plots of (A) wild-type *CNGA3I*, (B) mutant *CNGA3I*, (C) wild-type *CNGA3II*, (D) mutant *CNGA3II*, (E) wild-type *CNGA3III*, (F) mutant *CNGA3III*, and (G) the overlaid PCA plot of all systems.

**Fig 6:**
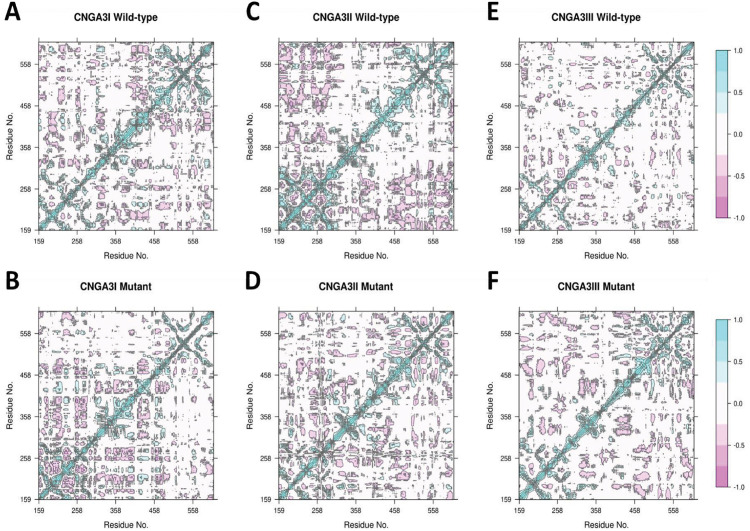
DCCM plots of (A) wild-type *CNGA3I*, (B) mutant *CNGA3I*, (C) wild-type *CNGA3II*, (D) mutant *CNGA3II*, (E) wild-type *CNGA3III*, and (F) mutant *CNGA3III.*

In family 4, the pathogenic variant was identified with duplication of a base pair, resulting in a premature protein termination at chr14:21,795,860dupT; c.2789dupT; p.Pro931Thrfs*3 in the *RPGRIP1* gene. Through co-segregation analysis, it was found that the affected individuals were homozygous for the mutation, while both parents were heterozygous carriers. The identified variant (c.2789dupT; p.Pro931Thrfs*3) in the *RPGRIP1* gene is predicted to lead to reduced function of the gene product due to premature termination after 934 amino acids, whereas the complete transcript is composed of 1,286 amino acids (Figs 1–6, Tables 1 and 2).

### Molecular dynamics simulation

The cryo-EM structure of human *CNGA3* (PDB ID: 7RHS) was used to investigate the effects of the Cys319Arg mutation. The mutation was introduced into all CNGA3 subunits (*CNGA3I, CNGA3II, CNGA3III*) of the *CNGA3/CNGB3* channel, which has C1 symmetry. Molecular dynamics (MD) simulations were performed on wild-type and mutant channels. Due to incomplete data in the cryo-EM structure, only residues Ile159–Leu611 were included in the analysis, omitting Ile612–Glu614 from *CNGA3I* and Ala158 from *CNGA3III*.

The root-mean-square deviation (RMSD) reflects protein structure fluctuation. After 20 ns, the mutant *CNGA3II* and *CNGA3III* showed higher RMSDs than the wild-type, with *CNGA3III* displaying the highest fluctuation. For *CNGA3I*, the RMSD difference was not significant. Root-mean-square fluctuation (RMSF) revealed greater fluctuation in the mutant *CNGA3III* across most domains. The radius of gyration (Rg) was highest for *CNGA3I* and lowest for *CNGA3III*, with the mutant *CNGA3II* exhibiting a higher Rg than its wild-type counterpart. The solvent-accessible surface area (SASA) was higher for mutants in *CNGA3II* and *CNGA3III*, suggesting increased solvent exposure, while *CNGA3I* showed a lower SASA for the mutant. Analysis of hydrogen bonds (H-bonds) indicated a slight decrease in the number of H-bonds in the mutant subunits, suggesting protein destabilization, though the changes were not highly significant.

To further investigate the effect of the Cys319Arg mutation on *CNGA3*, secondary structures of the wild-type and mutant subunits were analyzed based on the average structure calculated from frames after 20 ns of simulations. The mutation generally decreased the alpha helix content across all subunits, increasing coil and 3/10 helix content. For *CNGA3I* and *CNGA3II*, there was also a disruption in the beta sheet and an increase in turns. Specific protein regions were affected, including the disruption of the alpha helix in the Tyr184–Met204 region, where the gap in the wild-type structure is enlarged and filled with a 3/10 helix in the mutants. For *CNGA3III*, the disruption was more pronounced. Other regions such as Gln248–Leu254, Asp260–Val266, Pro298–Leu306, Ser401, and Glu455–Leu457 also showed changes in secondary structure, with alpha helices being replaced by 3/10 helices or vice versa. These structural disruptions suggest a destabilizing effect of the mutation on the *CNGA3* protein.

During MD simulations, principal component analysis (PCA) was performed to evaluate the conformational changes of the *CNGA3* subunits (wild-type and mutant). For *CNGA3I*, the wild-type conformation shifted from the center of the PCA plot to the lower-left corner, while the mutant stayed near the center. For *CNGA3II,* the conformational changes were minimal, with both wild-type and mutant subunits exploring similar regions in the PCA plot. The most significant difference was observed in *CNGA3III,* where the wild-type conformation moved upwards, and the mutant shifted to the lower-right corner. The PCA plot showed minimal overlap between wild-type and mutant *CNGA3III*, suggesting distinct conformations. In contrast, *CNGA3I* exhibited some overlap, indicating similarity between wild-type and mutant conformations, while *CNGA3II* showed considerable overlap, suggesting minimal conformational difference between the two.

We analyzed the dynamic cross-correlation matrix (DCCM) for the wild-type and mutant subunits of *CNGA3*. For *CNGA3I*, the wild-type showed mostly negative correlations across the entire protein, while the mutant concentrated its correlations at the N-terminal. In *CNGA3II*, the wild-type displayed positive correlations near the N-terminal and negative correlations between the N-terminal and C-terminal regions. However, these patterns were altered in the mutant, where the positive correlations disappeared. For *CNGA3III*, the wild-type had small patches of negative correlations throughout, but these patches were more prominent and more pronounced in the mutant. The differences in the DCCM suggest that the Cys319Arg mutation impacts the motion of almost all residues, either disrupting or enhancing their correlated and anti-correlated movements.

## Discussion and conclusion

Achromatopsia (ACHM; MIM #216900) is a rare, hereditary, autosomal recessive condition characterized by color blindness, photophobia, reduced visual acuity, and nystagmus. It has a prevalence of 1 in 30,000 and can present in complete or incomplete forms, with incomplete being less severe than complete [4]. The *CNGA3* gene (OMIM600053) encodes the alpha subunit of the CNG ion channel, which mediates ion permeation, especially calcium, through the photoreceptor cell membrane upon activation by light stimuli [5]. The *CNGA3* protein consists of six transmembrane helices (S1 to S2), a C-linker that connects the pore region, and a cyclic nucleotide binding domain. It has 694 amino acids, a molecular weight of 78.8 kDa, and eight coding exons [6]. The c.955T > C variant (p.Cys319Arg) has been implicated in ACHM, characterized by color blindness, nystagmus, and decreased visual acuity. This variant has been described in patients with similar phenotypes, further supporting its pathogenic role in disrupting normal cone cell function. Two families in our cohort demonstrated mutations in *CNGA3*, with a p.Cys319Arg missense mutation co-segregating successfully in the Family 1 cohort and a frameshift mutation (c.1443dup; p.Ile482Hisfs*6) identified in Family 2. Nystagmus was reported to develop or worsen at that age, consistent with previous reports that frameshift mutations in the *CNGA3* gene severely impair cone photoreceptor function, leading to achromatopsia alongside nystagmus. The identification of these variants reinforces the importance of *CNGA3* in maintaining visual function. Over 150 mutations involving *CNGA3* associated with ACHM have been reported in the HGMD database [23]. We followed up the same family with congenital stationary night blindness (CSNB). CSNB is inherited in an X-linked pattern and mutations in the *CACNA1F* gene (OMIM: 300,110) account for approximately 55% of CSNB cases [23]. It is also implicated in X-linked cone-rod dystrophy. The *CACNA1F* gene provides instructions for making a voltage-gated calcium channel essential for signals from photoreceptors to the inner retina. CSNB is characterized by nystagmus, poor visual acuity, nyctalopia, and defective color vision. In our study, Family 3 had a novel missense mutation (c.2254G > A; p.Val752Met) co-segregating with nystagmus, deficient vision, and color vision defects in the individuals studied in *CACNA1F*. The c.2254G > A variant, associated with a spectrum of retinal dystrophies, is known to affect the function of voltage-gated calcium channels, interrupting synaptic transmission in photoreceptors. This variant has also been identified in other populations, emphasizing its importance in retinal pathophysiology. Leber congenital amaurosis (LCA) type 6, a severe retinal dystrophy that often causes early vision loss and nystagmus, has recently been attributed to the *RPGRIP1* gene. Family 4 presented a new frameshift mutation (c.2789dupT, p.Pro931Thrfs*3) in the *RPGRIP1* gene, leading to truncation of a 1286-amino acid protein. The mutation is not found in gnomAD, and 161 mutations in the *RPGRIP1* gene have been reported to disrupt protein function. Mutations in *RPGRIP1* cause severe retinal degeneration and are typically associated with early-onset vision loss. *RPGRIP1* enables the transport of proteins and other molecules required by photoreceptors by maintaining the structure of their connecting cilium, making it vital for photoreceptor function [2]. The identification of this new mutation is supported by *RPGRIP1*’s established importance in retinal preservation and function. While the harmfulness of the variants in this study was evaluated according to ACMG guidelines, the missense mutation (p.Cys319Arg) and frameshift mutation (p.Ile482Hisfs*6) in *CNGA3* are both highly likely to be pathogenic due to their far-reaching disruption to cone cell function (based on segregation in affected families and previously reported variants). For *CACNA1F,* p.Val752Met is likely pathogenic, owing to its conservation across species, contribution to calcium channel dysfunction, and consistent association with retinal dystrophies. The frameshift mutation in *RPGRIP1* (p.Pro931Thrfs*3) was classified as likely pathogenic as it leads to premature truncation of the protein and disrupts its functional role in photoreceptor maintenance. The absence of this variant in population databases supports its pathogenicity. More stringent evidence that these mutations directly affect protein function would be functional validation, such as mRNA analysis or protein assays. Herein, we employed molecular dynamics (MD) in this study to evaluate how the Cys319Arg substitution mutation affects the *CNGA3* protein. Simulations showed significant variations in fluctuation, compactness, and a decrease in hydrogen bonds between carboxyl groups of two amino acids, indicating potential destabilization of the protein. Like other mutations in *CNGA3*, a disruption in the secondary structure, especially in the alpha helix, was observed in all three subunits, which could be detrimental to protein function. Principal component analysis (PCA) revealed marked differences in the conformation of the wild-type and mutant proteins, with the most notable differences found for *CNGA3III*, further supporting that the mutation plays a role in modulating protein dynamics. Dynamic cross-correlation matrix (DCCM) analysis suggests that the mutation modifies the motion of many residues within the whole protein at once, thereby affecting the channel’s functionality. These data indicate that the Cys319Arg mutation affects the structure and function of *CNGA3,* resulting in the clinical symptoms observed here. This study has several limitations. The small sample size means that our results cannot necessarily be generalized, and there was no functional confirmation of some variants, limiting our ability to classify them as pathogenic definitively. Phenotypic heterogeneity also complicates genotype-phenotype correlations, and clinical manifestations can vary considerably between patients with the same mutation. Exome analyses can also miss significant regulatory and intronic variants that may contribute to the development of the disease, as these act outside the protein-coding regions of the genome. Additionally, we focused our analysis on single-nucleotide variants (SNVs) and small indels and have not described any potential structural variants (large deletions or duplications) that may have occurred.

In conclusion, this study identified four likely pathogenic variants in *CNGA3, CACNA1F*, and *RPGRIP1* in four unrelated consanguineous Pakistani families, contributing to our understanding of the genetic basis of retinal dystrophies. These findings pave the way for improved genetic counseling and may offer insights into potential targeted therapies for individuals affected by conditions like nystagmus and associated visual impairments.
